# Modification of heat-related effects on mortality by air pollution concentration, at small-area level, in the Attica prefecture, Greece

**DOI:** 10.1186/s12940-024-01053-7

**Published:** 2024-01-24

**Authors:** Sofia Zafeiratou, Evangelia Samoli, Antonis Analitis, Konstantina Dimakopoulou, Christos Giannakopoulos, Konstantinos V. Varotsos, Alexandra Schneider, Massimo Stafoggia, Kristin Aunan, Klea Katsouyanni

**Affiliations:** 1 Department of Hygiene, Epidemiology and Medical Statistics, Medical School, National and Kapodistrian University of Athens, Athens, Greece; 2 Institute for Environmental Research and Sustainable Development, National Observatory of Athens, Athens, Greece; 3 Institute of Epidemiology, Helmholtz Zentrum München (HMGU), Neuherberg, Germany; 4 Department of Epidemiology of the Lazio Region Health Service (ASL ROMA 1), Rome, Italy; 5 CICERO Center for International Climate Research, Oslo, Norway; 6 Environmental Research Group, MRC Centre for Environment and Health, Imperial College London, London, UK

**Keywords:** Mortality, Air temperature, Air pollution, Effect modification

## Abstract

**Background:**

The independent effects of short-term exposure to increased air temperature and air pollution on mortality are well-documented. There is some evidence indicating that elevated concentrations of air pollutants may lead to increased heat-related mortality, but this evidence is not consistent. Most of these effects have been documented through time-series studies using city-wide data, rather than at a finer spatial level. In our study, we examined the possible modification of the heat effects on total and cause-specific mortality by air pollution at municipality level in the Attica region, Greece, during the warm period of the years 2000 to 2016.

**Methods:**

A municipality-specific over-dispersed Poisson regression model during the warm season (May–September) was used to investigate the heat effects on mortality and their modification by air pollution. We used the two-day average of the daily mean temperature and daily mean PM_10_, NO_2_ and 8 hour-max ozone (O_3_), derived from models, in each municipality as exposures. A bivariate tensor smoother was applied for temperature and each pollutant alternatively, by municipality. Α random-effects meta-analysis was used to obtain pooled estimates of the heat effects at different pollution levels. Heterogeneity of the between-levels differences of the heat effects was evaluated with a Q-test.

**Results:**

A rise in mean temperature from the 75th to the 99th percentile of the municipality-specific temperature distribution resulted in an increase in total mortality of 12.4% (95% Confidence Interval (CI):7.76–17.24) on low PM_10_ days, and 21.25% (95% CI: 17.83–24.76) on high PM_10_ days. The increase on mortality was 10.09% (95% CI: − 5.62- 28.41) on low ozone days, and 14.95% (95% CI: 10.79–19.27) on high ozone days. For cause-specific mortality an increasing trend of the heat effects with increasing PM_10_ and ozone levels was also observed. An inconsistent pattern was observed for the modification of the heat effects by NO_2_, with higher heat effects estimated in the lower level of the pollutant.

**Conclusions:**

Our results support the evidence of elevated heat effects on mortality at higher levels of PM_10_ and 8 h max O_3._ Under climate change, any policy targeted at lowering air pollution levels will yield significant public health benefits.

**Supplementary Information:**

The online version contains supplementary material available at 10.1186/s12940-024-01053-7.

## Introduction

The adverse effects of the short-term exposure to increased air temperature, as well as increased particulate and gaseous pollutants concentrations, on all-cause and cause-specific mortality are well established [1–10].

It is plausible to hypothesize and explore possible synergistic effects of elevated temperature and increased air pollutant concentrations. Air pollutant concentrations are affected by meteorological conditions [11–13], through chemical reactions, transport patterns and boundary layer height affecting the vertical mixing of pollutants. Furthermore, effect modification of temperature-related mortality by air pollution is biologically plausible, as both environmental exposures share common pathophysiological pathways, including oxidative stress and inflammatory response that may be enhanced by a joint exposure resulting in synergistic effects [14–17]. In the context of climate change and global warming, air temperature is expected to increase until the end of the century under all climatic scenarios [18], whereas air pollution can be reduced within a decade if suitable control measures are implemented [19–21]. Hence, the identification of possible effect modification of temperature-related mortality by air pollution may be of great importance for public health benefits [22].

Several systematic reviews and multilocation studies have investigated modification of the temperature effects on mortality and, similarly, modification of pollution effects by temperature levels reporting modification. The systematic review of Anenberg et al. [23] reported the synergistic all-cause mortality and cause- specific mortality effects of air pollution and heat (particularly for ozone (O_3_) and particulate matter (PM)), while Hu et al. [24] found statistically significant higher heat effect on all-cause and non-accidental mortality for increased levels of particulate matter with an aerodynamic diameter of < 10 μm (PM_10_) and O_3_. Chen et al. [22] reported stronger associations between high temperatures and total mortality at high PM (24 h mean), and O_3_ (maximum 8-hour moving average) levels, as well as between elevated pollution levels and total mortality at high compared to low temperatures. The EuroHEAT project [25] observed stronger heat wave effects on total and cardiovascular mortality on days with elevated concentrations of PM_10_ and O_3_. The PHASE project [26] reported an increase in all-cause mortality with higher levels of ozone and in cardiovascular mortality with higher levels of PM_10_ in European cities. No statistically significant effect modification was identified for effects on respiratory mortality, although the heat effect itself was statistically significant and stronger than the other mortality causes. The recent study conducted by Stafoggia et al. [27] in 640 cities worldwide also supported the interactive effects of air temperature and PM_10_ or O_3_ on all-cause mortality during the warm period, while slightly weaker results were found for the interactive effects of PM_2.5_ and NO_2_ with air temperature. The other recent large-scale multi-city multi-country study on cardiorespiratory mortality by Rai et al. [16] examined NO_2_ and PM_2.5_ as effect modifiers among others, for which the evidence so far has been scarce, and reported higher heat-related effects at increased pollutant levels, for all pollutants under investigation.

The majority of the above effect modification studies have been implemented mostly based on time-series studies of city-wide data [1, 2]. An analysis at a finer spatial level, such as the municipalities within a larger area, can provide a more accurate exposure definition for the study area and could reveal exposure and/or effect contrasts, especially among areas with varying population density and degree of urbanicity. The investigation of the temperature effects on mortality at a small area level, and especially their modification by air pollutant levels, can be of great importance as differential risks can be identified areas characterized to a larger extent by the urban heat island effect and between more and less polluted areas within a larger metropolitan region [28].

Within the context of the European project “Exposure to heat and air pollution in EUrope –cardiopulmonary impacts and benefits of mitigation and adaptation” (EXHAUSTION) we present here results on the effects of the short-term exposure to heat on total and cause-specific mortality, as well as their modification by air pollution, in the Attica-NUTS 2 region in Greece, subdivided at municipality level.

## Data and methods

### Study area

The Attica region comprises about 35% of the national population, i.e. 3.81 million inhabitants according to the 2011 census, where almost 84% of this population lives in the Greater Athens Metropolitan Area. The study area is divided into 66 municipalities, of which 42 belong to the Greater Athens Metropolitan Area. We excluded four municipalities that are at a large distance from the main Attica region, but belong to Attica for traditional administrative reasons (representing some of the islands, such as Kythira) and five municipalities (at the boundary of the study area) due to the lack of air pollution data. The average population of the municipalities was 65,123 inhabitants, ranging from 13,056 (municipality of Aegina) to 664,046 (municipality of Athens).

### Meteorological and air pollution data

Data on daily mean air temperature for the Attica region were provided by the National Observatory of Athens at high spatial resolution of 1 km × 1 km for the years 2000–2016. Specifically, daily mean temperatures were obtained for the corresponding period from the MESCAN-SURFEX reanalysis data set [29]. This dataset has a horizontal resolution of about 5.5 km × 5.5 km, a six-hourly temporal resolution and covers the period 1961–2019. Subsequently, the six-hourly air temperature at 2 m were post-processed to obtain the daily means. The methodology followed is similar to that reported in Varotsos et al. (2023) [30] where the authors transferred the spatial variability of Weather Research and Forecasting (WRF) Model over Attica to an observational daily gridded data set. In particular, in the present study the following steps have been implemented:

i) Initially a Principal Components Analysis (PCA) was performed on the daily WRF data to identify areas with homogeneous characteristics for all the variables examined in the study. PCA was performed for each of the months of 1995 and the number of retained components depended on the percentage of the total variability explained by these principal components. The target was to explain the 99% of the total variability of temperature. The mean monthly annual cycles over the period 2000–2016 were calculated for the regridded MESCAN-SURFEX. Consequently, for the WRF grid points with the highest loadings in each one of PCs the monthly means were calculated for the year 1995.
ii) The biases (absolute) of the monthly means between the closest MESCAN-SURFEX grid points to the WRF grid points with the highest loadings were interpolated on the 1 km WRF grid using Ordinary Kriging and then added to the WRF monthly means output. This local perturbation was performed in order to obtain a WRF product with climate temporal characteristics similar to MESCAN-SURFEX that will still maintain the spatial variability of WRF.
iii) The final step to obtain the 1km × 1km statistically downscaled MESCAN-SURFEX variables was to transfer the WRF spatial variability to the regridded daily datasets. This was achieved by using the unbiasing bias adjustment method [31]. In particular, the mean monthly deviations (absolute) between the perturbed WRF simulation and the mean monthly regridded values for the 17-year period (2000–2016) for each grid point were calculated. Subsequently, to obtain the final daily gridded data, the mean monthly deviations were added to the daily regridded MESCAN-SURFEX values. This method maintains the absolute trend as well as the temporal variability of the original dataset. For the purposes of the study the values at the center of each grid were calculated.

Data on air pollution included modelled concentrations of 24 h PM_10_, 8 h max O_3_ and 24 h nitrogen dioxide (NO_2_). Spatio-temporal land use regression (LUR) models were used to estimate the concentrations of the pollutants. A semiparametric approach including linear and smooth functions of spatial and temporal covariates and a bivariate smooth thin plate function for the geographical coordinates (longitude, latitude) of fixed monitoring stations was applied. The LUR models for PM_10_ and NO_2_ included the traffic load in a 100 m buffer around monitoring stations, the daily mean temperature, relative humidity, wind speed and wind direction measured from a fixed station and the presence of a day with Sahara dust, only for PM_10_ model, as predictor variables. The LUR models for O_3_ included the daily mean temperature, relative humidity, wind speed, wind direction, cloud coverage and solar radiation between 4:00 pm and 7:00 pm measured from a fixed station, the urban green area in a buffer of 300 m around the monitoring stations, the major road length in a buffer of 500 m around the monitoring stations and the traffic intensity on the nearest major road as predictor variables. These models had previously been developed for the Greater Athens Area that comprises about half of the study area (from year 1997 for NO_2_ & 2001 for PM_10_ & O_3_) [32, 33] and were updated until 31st December 2016 and extended spatially over the area of Attica for use in EXHAUSTION. The developed models explain the 75, 53 and 76% of the spatio-temporal variation for NO_2_, PM_10_ and O_3_, respectively. The daily concentrations of 24 h PM_10_, 8 h max O_3_ and 24 h NO_2_ were predicted by the LUR models at the same location points (grid centers) that the temperature data were provided.

Geographic Information Systems (GIS) were used to identify the grid-center points that were within the boundaries of each municipality. To calculate temperature values and air pollution concentrations at municipality level, the exposure estimates at the points that fell inside the boundaries of each municipality were averaged.

### Mortality data

We obtained aggregated data on daily counts of mortality from natural (International Classification of Diseases (ICD)-10: A00 - R99), cardiovascular (ICD10: I00-I99) and respiratory causes (ICD-10: J00 - J99) at municipality level for the years 2000–2016, from the Hellenic Statistical Authority (ELSTAT). Deaths due to cardiopulmonary disease were considered as the total number of deaths resulting from cardiovascular and respiratory causes.

### Statistical methods

Descriptive statistics are given for the total set of all daily observations i.e. *n* = 57 municipalities by 2,601 days.

### Heat effects on mortality

The effect of high air temperature on mortality outcomes was assessed in two stages. First, an over-dispersed Poisson regression model was applied for each municipality, for the warm period of the year, defined as the five-month period from May to September, as shown in (1):

1 $$\mathit{\log}\left[E\left({Y}_i\right)\right]=\alpha +s\left({Tmean}_{0-1}\right)+\kern0.5em s(ytrend)+ dow$$

where *Y*_*i*_ is the number of deaths in the day *i*; *s*(*Tmean*_0 − 1_) is a non-parametric smoothing spline with 2 degrees of freedom (df) for the two-day moving average (lags 0–1) of the same and the previous day air temperature; *s*(*ytrend*) is a natural spline with 3 df per year for the time trend, and *dow* is the day of the week.

The heat effect was estimated as the percent change in mortality (and the corresponding 95% confidence interval (CI)) for an increase in mean temperature from the 75th to the 99th percentile of the municipality-specific daily mean temperature distribution.

At the second stage, the municipality-specific effect estimates were pooled using a random-effects meta-analytical model to assess the overall heat effect on mortality in the Attica region. The sensitivity of our findings was assessed by 1) using lags 0–3 for temperature and 2) restricting the warm period to the 3 warmest months (June–August). We also estimated the heat effects corresponding to an increase in mean temperature from the Minimum Mortality Temperature (MMT) - the temperature value where the minimum mortality is observed- to the 99th percentile of the municipality-specific distribution.

### Modification of heat effects by air pollution

The two-stage approach was also applied to assess the interactive effects between temperature and air pollution on mortality. Again, as a first stage, an over-dispersed Poisson regression model in each municipality was used, as shown in (2):

2 $$\mathit{\log}\left[E\left({Y}_i\right)\right]=\alpha + tensor\left({Tmean}_{0-1},{Pollutant}_{0-1}\right)+\kern0.5em s(ytrend)+ dow$$

where *Y*_*i*_, *Tmean*_0 − 1_, *s*(*ytrend*) and *dow* as defined in eq. (1) and *Pollutant*_0 − 1_ is the 2-day moving average of the air pollutant considered (PM_10_, O_3_ or NO_2_).

The interaction between temperature and air pollution was included in the model as a tensor smoother that allows to model the combined relationship between two risk factors and the outcome of interest with a non-parametric approach, by running a “response surface” model [17]. As a result, we get a tridimensional curve modeling the increases in mortality according to a combined variation of the values of temperature and air pollutant. In our model the tensor was estimated by fitting a cubic spline with 3 df for both temperature and each pollutant alternatively.

To quantify the interactive effects of temperature and pollutants on mortality, we estimated the temperature effect at three levels of air pollution, considered as “low”, “medium”, and “high” levels, corresponding to the 5th, 50th, and 95th percentile of the municipality-specific air pollutants distributions.

For each level of air pollution, the heat effect was estimated as the percent change in mortality for an increase in mean temperature from the 75th to the 99th percentile of the municipality-specific distribution, in order to capture the effects of the extreme heat. Again, a random-effects meta-analytical model was applied to assess the overall heat effect on mortality in Attica region, for the three air pollution levels. Differences of the heat effects between the different air pollution levels were assessed through a Q-Test for between subgroup heterogeneity.

We applied the following sensitivity analyses: first, we changed the lag from 0-1 to 0–3 (for both air temperature and air pollutants); second, we defined “low” and “high” levels of air pollutants as the 25th and 75th percentiles (rather than the 5th and 95th); third, we restricted the “warm” season to the three warmest months June–August (instead of May–September); fourth, we restricted the analysis to the most populated municipalities (those with population above 43,282 inhabitants (median population of municipalities)).

All analyses were carried out using R software (version 4.2.1) with libraries *gnm, dlnm*, and *mixmeta*.

## Results

The distribution of mortality outcomes is presented in Table 1. A total number of 229,315 deaths from natural causes occurred within the study period, with 43.8% of them due to cardiovascular causes and 10.2% due to respiratory causes. The range of the daily number of deaths is wide, reflecting the different size of population in the different municipalities. Detailed descriptive statistics for the 57 municipalities are presented in the Supplement (Table S1).

**Table 1:** Descriptive characteristics of daily mortality by municipality overall Attica region in 2000–2016

	Mean (sd)	Range
**Mortality causes**		
**Natural**	1.50 (2.93)	0–48
**Cardiopulmonary**	0.81 (1.73)	0–35
**Cardiovascular**	0.66 (1.44)	0–28
**Respiratory**	0.15 (0.49)	0–8

**Fig. 1:**
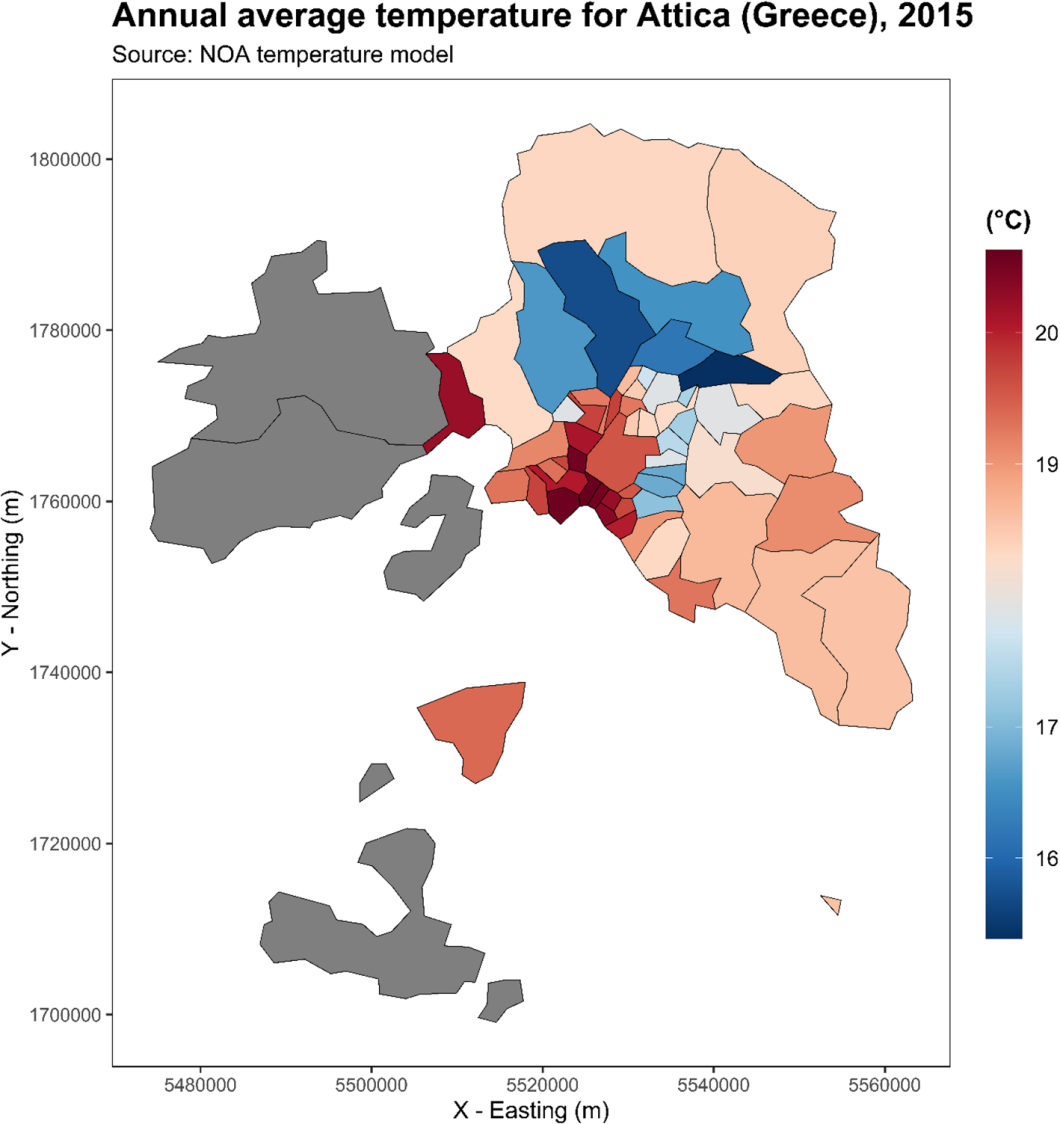
Warm period (May to September) mean air temperature by municipality at the Attica region, Greece for the year 2015

**Fig. 2:**
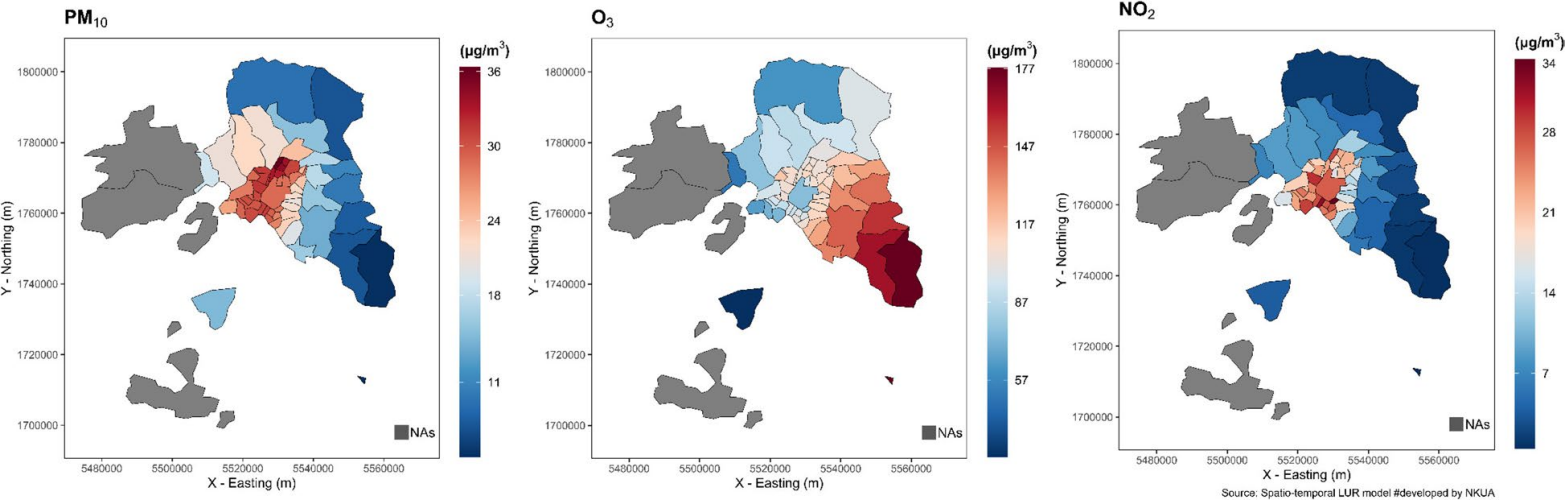
Warm period (May to September) mean concentrations of PM10 (left), 8 h max O3 (middle) and NO2 (right), by municipality at the Attica region, Greece for the year 2015

Table 2 shows descriptive statistics for air temperature and air pollutants. The average daily temperature of the warm period for the years 2000–2016 was 25.0 °C, with daily ranges from 10.3 °C to 40.7 °C. Detailed descriptive statistics of daily temperature for the 57 municipalities are reported also in Table S1 of the Supplement. On average, daily concentrations of PM_10_, O_3_ and NO_2_ were 30.6 μg/m^3^, 102.9 μg/m^3^ and 21.8 μg/m^3^, respectively. In general, the municipalities at the central and south-west of the Attica presented higher temperatures by as much as 4 °C compared to those in the north (Fig. 1). The same municipalities at the center and south of the study area had higher levels of PM_10_ and NO_2_, while municipalities at east Attica, outside the metropolitan area, had higher levels of O_3_ (Fig. 2).

**Table 2:** Descriptive characteristics of daily estimated temperature values and air pollution concentrations by municipality overall Attica region in 2000–2016

	Mean (sd)	Minimum	5th percentile	25th percentile	50th percentile	75th percentile	95th percentile	99th percentile	Maximum
**Temperature** (°C)	25.03 (3.93)	10.28	18.22	22.14	25.55	28.04	30.72	32.31	40.68
**Air pollutants**									
**PM**_**10**_ (24 h μg/m^3^)^a^	30.65 (13.81)	2.75	9.31	21.03	29.70	39.19	54.58	68.54	128.38
**O**_**3**_ (8h max μg/m^3^) ^a^	102.93 (27.71)	0.10	56.62	88.07	103.53	117.00	151.58	177.55	204.79
**NO**_**2**_ (24 h μg/m^3^)	21.80 (14.96)	0.19	1.52	9.26	20.33	31.63	49.09	61.24	99.01

^a^Available data for 2001–2016

An increase in daily mean temperature from the 75th to the 99th municipality-specific percentile was associated with a pooled increase of 12.78% (95% Confidence Interval (CI): 11.12, 14.45%) in natural cause mortality, while the effect on cardiopulmonary mortality was of similar magnitude (13.38% increase (95% CI: 10.83, 15.99%)) (Table 3). The heat effect was higher for respiratory mortality (22.62% increase (95% CI: 16.85, 28.68%)) (Table 3). Comparable effect estimates were observed in all sensitivity analyses, except for the effect on cardiovascular mortality, that was lower when we considered the lagged effect of temperature up to 3 days (4.36% increase (95% CI: 2.28, 6.47%)) (Table S2). However, a larger effect on mortality was estimated for an increase in daily mean temperature from the MMT to the 99th municipality-specific percentile, as this value is much lower than the 75th temperature percentile of each municipality, used in the main approach.

**Table 3:** Percent change (and 95% Confidence Interval (CI)) in mortality per increase in the 2-day mean temperature from the 75th to the 99th percentile of the area-specific distribution, (a) without an interaction with air pollution and (b) by low, medium, and high levels of air pollutant represented by 5th (l), 50th (m), and 95th (h) percentile of area-specific distribution. Results of second stage analysis pooling municipality level effects^a^

Pollutant level	Natural-cause mortality	*p*-value^b^	Cardio-pulmonary mortality	*p*-value^b^	Cardiovascular mortality	*p*-value^b^	Respiratory mortality	*p*-value^b^
**No pollutant**								
–	12.78 (11.12–14.45)		13.38 (10.83- 15.99)		9.97 (7.31- 12.70)		22.62 (16.85- 28.68)	
**PM**_**10**_								
l	12.40 (7.76–17.24)	0.012	5.78 (−0.96–12.98)	< 0.001	1.08 (− 5.41–8.01)	0.001	7.72 (− 7.04–24.82)	< 0.001
m	19.54 (16.64–22.50)		14.54 (11.20–17.98)		7.42 (4.04–10.92)		40.91 (30.47–52.17)	
h	21.25 (17.83–24.76)		23.47 (18.72–28.41)		15.98 (11.16–21.00)		54.30 (38.97–71.31)	
**O**_**3**_								
l	10.09 (−5.62–28.41)	0.450	− 7.84 (− 23.48–10.99)	0.001	−7.61 (− 27.01–16.94)	0.053	25.61 (−12.72–80.77)	0.008
m	11.79 (9.00–14.64)		11.55 (6.92–16.39)		8.87 (3.72–14.28)		12.57 (1.12–25.32)	
h	14.95 (10.79–19.27)		23.66 (16.61–31.13)		17.72 (10.06–25.91)		47.69 (29.34–68.64)	
**NO**_**2**_								
l	11.00 (7.60–14.51)	< 0.001	8.94 (3.92–14.20)	< 0.001	6.47 (0.43–12.87)	0.040	15.56 (4.97–27.21)	0.019
m	15.29 (13.23–17.39)		16.90 (13.90–19.99)		13.24 (10.01–16.56)		27.58 (19.00–36.78)	
h	5.78 (2.50–9.17)		7.35 (3.39–11.46)		6.04 (0.40–12.00)		4.75 (−7.83–19.04)	

^a^Pooled estimates from municipality-specific over-dispersed Poisson regression models, including (a) a smoothing spline with 2 df for the two-day moving average of air temperature and (b) a tensor smoother for the two-day moving averages of both air temperature and air pollutant, adjusted for seasonal trends and day of the week

^**b**^*p-value from Q-test for heterogeneity between subgroups*

A clear increase in heat effect was observed for increasing PM_10_ levels: 12.4% (95% CI: 7.76, 17.24%) increase in natural cause mortality due to heat was estimated during days with low PM_10_ levels, which increased up to 21.25% (95% CI: 17.83, 24.76%) during high PM_10_ days (Table 3). A similar pattern was observed for cardiopulmonary mortality, with the increase in high PM_10_ days to be over 23%, and the heat effect on low PM_10_ days being much smaller and not statistically significant (Table 3). The most pronounced increase was for respiratory mortality for all levels of PM_10_ (in high PM_10_ days to be 54.30% (95% CI: 38.97, 71.31%)) (Table 3). Statistically significant differences on the 5% significance level were observed between the different pollutant levels for all the causes of death (Table 3).

A similar pattern was observed for O_3_ levels, with non-statistically significant effects of heat on low ozone days, and mortality increases on high ozone days that ranged from 14.95% (95% CI: 10.79, 19.27%) for natural cause to 47.69% (95% CI: 29.34, 68.64%) for respiratory mortality (Table 3). For O_3_, statistically significant differences between the different levels were observed for cardiopulmonary, and respiratory mortality (*p*-value< 0.05), while suggestive heterogeneity was present for cardiovascular mortality (*p*-value = 0.053) (Table 3).

Inconsistent results were found for the modification of the heat effects on mortality by NO_2_. Higher increases on mortality were found for days with medium levels of NO_2_, while for days with high NO_2_ levels the heat effects were decreased or non-statistically significant (Table 3). Again, the higher increase in mortality associated with temperature was estimated for respiratory mortality (15.56% increase (95% CI: 4.97, 27.21%) on low NO_2_ days and 27.58% increase (95% CI: 19.00, 36.78%) on medium NO_2_ days) while the smallest increase was found for cardiovascular mortality (6.47% increase (95% CI: 0.43, 12.87%) on low NO_2_ days and 13.24% increase (95% CI: 10.01, 16.56%) on medium NO_2_ days). However, the differences between the levels of NO_2_ were statistically significant on the 5% significance level (Table 3).

Results of sensitivity analyses were generally robust when considering a different definition for the warm period and different exposure windows for temperature and air pollutants (Table S3) or when using the 25th and the 75th percentile for the definition of low and high air pollution levels respectively. Sub-analyses included only the most populated municipalities also provided consistent results (Table S3).

## Discussion

Our study is among the few assessing the modification of air temperature effects on natural and cause-specific mortality by air pollution in the warm season, at a small spatial scale within a larger region. Considering the variable levels of exposure within the study area, we estimated a consistent overall increasing trend of the heat effects on mortality with increasing levels of PM_10_ and O_3_, while the modification of heat effects by NO_2_ was not consistent.

Previous studies on heat-mortality effects focusing on natural and cause-specific mortality have mainly used city-wide rather than small-area level data. Our findings support previous evidence regarding the effects of increased air temperature on mortality [1, 2, 5, 34–37], with more pronounced effects for respiratory mortality [1, 2, 5, 34]. Our results are also in agreement with studies taking into account spatial differences of the exposure within larger areas [5, 34].

Consistent results have been found for the synergy between increased air temperature and PM_10_. A study in nine European countries [26] found increasing heat effects on natural-cause and cardiovascular mortality, reporting a statistically significant interaction for cardiovascular mortality, whilst inconsistent results were found for respiratory mortality. A study in Italy [17], using a similar methodology to our study, supported that heat-related mortality was modified by PM_10_ levels. In cities in the south of Italy that share similar climatic conditions with our study area the heat-related mortality risk ranged from 7.5 to 21.6% in low and high PM_10_ days, respectively, which are of similar magnitude to our findings. A recent meta-analysis [24] found significant modification effects by PM_10_ on the association between heat and natural-cause mortality, with an increase on mortality from 6.3% (95% CI: 4.80, 7.80%) in low pollution levels to 11.4% (95% CI: 8.70,14.20%) in high pollution levels, while similar results are reported in the large-scale study including 640 cities (5.3% (95% CI: 3.8, 6.9%) and 12.8% (95% CI: 8.7, 17.0%) increase on mortality when daily PM_10_ was equal to 10 or 90 μg/m^3^, respectively) [27]. For cardiovascular and respiratory mortality, a similar increasing pattern was identified, although the modification was not statistically significant. Finally, a global-scale study [16] on modification by air pollutants of the heat effects on cardiorespiratory mortality at city-level, reported significantly modified heat effect by PM_10_, with an increase in heat-related mortality on high PM_10_ days of 7.33% (95% CI: 7.29, 7.37%) and 14.62% (95% CI: 14.49, 14.74%) for cardiovascular and respiratory mortality, respectively, which are much lower than our estimates. As respiratory deaths represent smaller numbers, the inconsistent results may reflect reduced statistical power.

We found evidence of increased heat-related mortality risk at higher O_3_ levels, with non-statistically significant effects on low O_3_ days. Scortichini et al. [17] reported similar and slightly increasing heat-related effects on mortality with increasing O_3_ in the cities of the South of Italy. In a study in nine European cities, significantly higher effects were found for natural-cause mortality on high O_3_ days, while no effect modification was observed for cardiovascular and respiratory mortality [26]. The studies included in the recent meta-analysis of Hu et al. [24] reported that for natural-cause mortality, the increase was statistically more significant on high compared to low O_3_ days. The related increase on high pollution days was 12.5% (95% CI: 4.70, 20.90%), which is comparable to the estimate of our study. Stafoggia et al. [27] reported a 2.9% (95% CI: 1.1, 4.7%) increase on all-cause mortality when daily O_3_ concentrations were 40 μg/m^3^ and 12.5% (95% CI: 6.9, 18.5%) increase on mortality at elevated O_3_ concentrations (equal to 160 μg/m^3^). A similar finding was reported by Rai et al. [16] for cardiorespiratory mortality, where the risk for heat-related cardiovascular mortality increased from 1.60% (95% CI: 1.58, 1.61%) to 8.73% (95% CI: 8.69, 8.76%) and for respiratory mortality from 4.12% (95% CI: 4.09, 4.15%) to 13.53% (95% CI: 13.42, 13.65%) in days with low and high O_3_ levels, respectively.

PM_10_ concentrations increase on days with Sahara dust advection. To take this into account, we included days with Sahara dust advection as predictor in the PM_10_ LUR model. Increased O_3_ levels, that are observed on days with high air temperature, may be also caused by Sahara dust advection. In Southern Europe, where our study area is located, on days with Sahara dust, the O_3_ concentrations are lower than those on non-dust days and also Sahara-PM_10_ concentrations are negatively correlated with O_3_ concentrations [38]. Additionally, we limited our analysis from May to September, including mostly summer months, while in our study region days with dust episodes mainly occur on spring and fall [39]. So, we don’t expect any modification of the results if we additionally take into account advection of dust from the Sahara. Nevertheless, we further assessed the effect modification of the PM_10_-temperature association in the city of Athens (the urban center of our study area), as we do not expect any variability of Sahara dust advection between municipalities within Attica region, by including interaction terms in our smooth curve for levels above vs below the median O_3_ levels (results not shown) and the model did not present a better fit.

NO_2_ as an effect modifier of the heat effects on mortality has been less studied and the evidence is inconsistent [24]. No evidence for interaction between apparent temperature and NO_2_ was found in Analitis et al. [26], while the effects seemed to decrease in higher NO_2_ levels, which is consistent with the results of the present study. On the other hand, the global-scale multicity studies [16, 27] reported a statistically significant heat effect modification by NO_2_ on all-cause and cardiorespiratory mortality, with elevated levels of NO_2_ associated with higher heat-related effect, although the differences in the heat-effects between the different pollutant levels were not pronounced for cardiorespiratory mortality. However, differences in performance of the exposure assessment models in terms of the temporal component may be in part responsible for varying effects between pollutants. Also, considering that during daytime hours NO_2_ is converted to NO as a result of photolysis, which leads to O_3_ formation, an amount of NO_2_ is converted to O_3_, through various reactions [40] and this may partly explain the inconsistent results found on high NO_2_ days.

The interactive effects of temperature and air pollution on human health can be explained by several atmospheric chemistry, behavioral and biological pathways. Climate change affects the weather patterns and subsequently the concentration of pollutants, including O_3_, PM_10_ and NO_2_, as higher temperatures lead to O_3_ and secondary particle formation through chemical reactions. Additionally, in the warm period people spend more time outdoors and may thus be exposed to increased air pollution and heat. This increased exposure can potentially have adverse effects on health, particularly among more susceptible subgroups [41]. Higher temperature can increase the total inhalation and alter the composition and toxicity of air pollutants [14]. Prolonged heat exposure may cause reduced ability of the body to detoxify chemicals [14] and increased ventilation rate and lung volumes through thermoregulatory responses at the pulmonary level, resulting in enhancement of the overall intake of airborne pollutants [42]. Also, air pollutants and high temperature may share common pathophysiological pathways, including oxidative stress (especially for O_3_), inflammatory response, apoptosis, and growing levels of markers of systemic inflammation such as C-reactive protein (for PM) [17], while NO_2_ can cause damage to the lung cells directly [16].

Our study has the advantage of being one of the few studies exploring the effect modification of heat-related mortality by air pollution at a small area scale. We used fine-resolution modelled estimates by 1 km × 1 km for exposure assessment, which allowed us to represent the spatial variation of exposures within the study area. As a result, varying effect estimates of the different municipalities have been provided, leading to more accurate estimation of the effects of the wider area compared to an aggregation at city-wide level, as this approach takes into account the varying effects within the study area, along with their precision. Additionally, the study area covers not only the very densely populated Athens municipality but also less dense suburban areas, which are underrepresented in the current literature. Sensitivity analyses including the most populated municipalities, as a proxy of high degree of urbanicity, confirmed our results, reporting heat effects on mortality of similar magnitude and similar modification pattern by pollution levels. Another advantage of our study is the use of tridimensional non-parametric curves for the simultaneous modeling of non-linear associations between both exposures and mortality, which granted us greater flexibility.

Nevertheless, our study also has some limitations. First, we did not investigate the interactive effect of high temperatures and PM_2.5_, due to lack of PM_2.5_ modelled data in the study area, although there is ample evidence that this pollutant has adverse health effects [43]. Additionally, our study is limited to the Attica region that encompasses the country’s capital, which is a major urban area although some variability in the main urban characteristics is present, through the inclusion of suburban and few rural areas. Also, the population residing in the same municipality was assigned the same exposure to environmental factors, because of the lack of individual time-dependent exposure and behavioral data, being an inherent limitation in many air pollution epidemiology studies. Data on potential effect modifiers characterizing the housing conditions, which can mitigate the heat and air pollution exposures, were not examined in the present study. Finally, the expression of the effects on mortality for different pollution levels when considering a temperature increase from the 75th percentile, rather than the MMT, to the 99th percentile, may underestimate the effect as the MMT is in all cases much lower than the 75th percentile, as was observed in the main heat-related effect analysis on mortality. The choice to focus on the effects of extreme temperatures rather than the entire range from the MMT drove this decision.

Heat-health action plans (HHAPs) have been implemented in the recent years as an urgent need for the continuously increased number of heatwaves and extreme temperatures. Early warning systems have also been established to act on the risks posed by increased levels of air pollution. However, these plans are usually activated separately for each exposure, although the exposures are interrelated [44]. Some countries (Belgium, Hungary, Portugal, and Switzerland) focusing on extreme heat, also consider air pollution (primarily ozone) as a part of their environmental policies [45]. Our results indicate that an integrated action should be implemented, ensuring comprehensive protection of human health under the complex relationship between both environmental exposures and human health. Public health warning systems related to climate change should incorporate different indicators that reflect the existing relationship between different exposures and related health problems, thus protecting human health in an integral way [44].

## Conclusion

In 2021, the World Health Organization revised its Air Quality Guidelines, due to strong evidence for the air pollution-related health effects occurring at even lower concentrations than previously proposed. These guidelines are under discussion by the European Commission in 2023, under the revision process of existing air quality limits in EC. The EU’s strategy to reduce climate change impacts, through the elimination of black carbon emissions, is also expected to improve air quality [21]. As our research findings indicate that the heat-related effects on mortality are more pronounced at higher levels of PM_10_ while there is substantial evidence on more pronounced heat-related effects also on elevated concentrations of 8 h max O_3_, any EU policy targeting lowering air pollution levels will yield significant public health benefits, as it will mitigate the interactive effects on human health.

### Supplementary Information

**Additional file 1: Table S1.** Descriptive statistics of daily temperature, mortality and population density of the 57 municipalities, by population size category. **Table S2.** Heat effects as percent change (and 95% Confidence Interval (CI)) in outcome, for defined contrast in the 2-day mean temperature of each municipality. Results of second stage analysis pooling municipality level effects. **Table S3.** Percent change (and 95% Confidence Interval (CI)) in mortality per increase in the 2-day mean temperature from the 75^th^ to the 99^th^ percentile of the area-specific distribution, by low, medium, and high levels of air pollutant represented by 5^th^ (l), 50^th^ (m), and 95^th^ (h) percentile of area-specific distribution, unless it is stated otherwise - Sensitivity analyses.

## Data Availability

Data were obtained following a confidentiality agreement with local authorities and may not be shared. Access to coefficients from stage 1 models and R codes will be granted following a request to the researchers and the consortium.
