# Supplementary material for: Modification of heat-related effects on mortality by air pollution concentration, at small-area level, in the Attica prefecture, Greece
Source: Environ Health. 2024 Jan 24;23:10. doi: 10.1186/s12940-024-01053-7 (PMC10809516; doi:10.1186/s12940-024-01053-7)
Supplement: Supplementary file 1 — Additional file 1: Table S1. Descriptive statistics of daily temperature, mortality and population density of the 57 municipalities, by population size category. Table S2. Heat effects as percent change (and 95% Confidence Interval (CI)) in outcome, for defined contrast in the 2-day mean temperature of each municipality. Results of second stage analysis pooling municipality level effects. Table S3. Percent change (and 95% Confidence Interval (CI)) in mortality per increase in the 2-day mean temperature from the 75th to the 99th percentile of the area-specific distribution, by low, medium, and high levels of air pollutant represented by 5th (l), 50th (m), and 95th (h) percentile of area-specific distribution, unless it is stated otherwise - Sensitivity analyses. [file 12940_2024_1053_MOESM1_ESM.docx]

**Supplementary material**

**Table S1. Descriptive statistics of daily temperature, mortality and population density of the 57 municipalities, by population size category**

| **Population (inhabitants)** | **N** | **Population density** | **Daily temperature** | **Natural cause-mortality** | | **Cardiopulmonary mortality** | | **Cardiovascular mortality** | | **Respiratory mortality** | |
| --- | --- | --- | --- | --- | --- | --- | --- | --- | --- | --- | --- |
|  |  | **Mean (sd)** | **Mean (sd)** | **Mean (sd)** | **Range** | **Mean (sd)** | **Range** | **Mean (sd)** | **Range** | **Mean (sd)** | **Range** |
| **<20,000** | 3 | 264.29 (128.39) | 25.1 (3.67) | 0.35 (0.62) | 0-6 | 0.19 (0.45) | 0-5 | 0.15 (0.4) | 0-5 | 0.04 (0.21) | **0-3** |
| **20,000-39,999** | 20 | 3636.53 (4531.98) | 24.77 (3.93) | 0.62 (0.8) | 0-9 | 0.33 (0.58) | 0-7 | 0.27 (0.53) | 0-7 | 0.06 (0.25) | 0-3 |
| **40,000-59,999** | 11 | 4989.3 (3200.43) | 24.91 (3.91) | 0.9 (0.99) | 0-7 | 0.48 (0.71) | 0-5 | 0.39 (0.64) | 0-5 | 0.09 (0.3) | 0-3 |
| **60,000-79,999** | 14 | 10603.25 (5142.05) | 25.04 (3.93) | 1.52 (1.29) | 0-11 | 0.81 (0.93) | 0-7 | 0.65 (0.83) | 0-7 | 0.16 (0.4) | 0-5 |
| **80,000-99,999** | 4 | 6069.36 (4069.8) | 24.8 (4.13) | 1.71 (1.44) | 0-16 | 0.92 (1.03) | 0-12 | 0.76 (0.92) | 0-9 | 0.17 (0.42) | 0-3 |
| **100,000-499,999** | 4 | 15324.19 (4848.28) | 26.52 (3.59) | 3.51 (2.11) | 0-15 | 1.92 (1.49) | 0-14 | 1.57 (1.33) | 0-9 | 0.35 (0.61) | 0-5 |
| **>500,000** | 1 | 18640.35 (0) | 25.92 (3.79) | 20.16 (5.11) | 6-48 | 11.07 (3.86) | 1-35 | 8.89 (3.47) | 1-28 | 2.18 (1.53) | 0-8 |

**Table S2. Heat effects as percent change (and 95% Confidence Interval (CI)) in outcome, for defined contrast in the 2-day mean temperature of each municipality. Results of second stage analysis pooling municipality level effects^1^**

| **Model** | **Temperature contrast** | **Natural-cause mortality** | **Cardio-pulmonary mortality** | **Cardiovascular mortality** | **Respiratory mortality** |
| --- | --- | --- | --- | --- | --- |
| *Lag 0-1, May-September* | *99^th^ pct versus 75^th^ pct^2^* | 12.78 (11.12- 14.45) | 13.38 (10.83, 15.99) | 9.97 (7.31, 12.7) | 22.62 (16.85, 28.68) |
| *Lag 0-1, June-August* | *99^th^ pct versus 75^th^ pct^2^* | 10.74 (8.91, 12.6) | 11.25 (8.75, 13.82) | 8.16 (5.23, 11.17) | 23.77 (17.58, 30.27) |
| *Lag 0-3, May-September* | *99^th^ pct versus 75^th^ pct^2^* | 10.87 (9.16, 12.6) | 8.68 (6.54, 10.87) | 4.36 (2.28, 6.47) | 20.92 (15.79, 26.28) |
| *Lag 0-1, May-September* | *99^th^ pct versus MMT^2^* | 74.6 (63.23- 86.77) | 54.59 (44.16- 65.77) | 31.05 (23.37- 39.19) | 311.5 (228.26- 415.84) |
| ^1^Pooled estimates from municipality-specific over-dispersed Poisson regression models, including a smoothing spline with 2 df for the two-day moving average of air temperature, adjusted for seasonal trends and day of the week  ^2^pct: percentiles of the municipality-specific distribution  MMT: Minimum Mortality Temperature | | | | | |

**Table S3. Percent change (and 95% Confidence Interval (CI)) in mortality per increase in the 2-day mean temperature from the 75^th^ to the 99^th^ percentile of the area-specific distribution, by low, medium, and high levels of air pollutant represented by 5^th^ (l), 50^th^ (m), and 95^th^ (h) percentile of area-specific distribution, unless it is stated otherwise - Sensitivity analyses.**

| **Model** | **Pollutant level** | **Natural-cause mortality** | **Cardio-pulmonary mortality** | **Cardiovascular mortality** | **Respiratory mortality** |
| --- | --- | --- | --- | --- | --- |
| **PM_10_** (μg/m^3^) |  |  |  |  |  |
| *Different definition  of warm period (June-August)* | l | 10.24 (4.03- 16.83) | 3.3 (-3.71- 10.82) | -6.62 (-13.56- 0.87) | 33.58 (12.08- 59.2) |
|  | m | 19.33 (15.63- 23.14) | 14.64 (10.3- 19.15) | 6.67 (2.1- 11.45) | 51.57 (38.32- 66.08) |
|  | h | 18.79 (15.15- 22.55) | 23.06 (17.56- 28.82) | 18.81 (12.61- 25.36) | 46.44 (32.61- 61.71) |
| *Lag 0-3* | l | 11.7 (7.11- 16.47) | 1.98 (-3.54- 7.82) | -1.69 (-6.92- 3.84) | 5.43 (-8.63- 21.65) |
|  | m | 13.3 (10.88- 15.78) | 11.55 (8.63- 14.56) | 5.72 (2.88- 8.63) | 30.07 (21.34- 39.43) |
|  | h | 17.28 (13.96- 20.69) | 20.17 (14.94- 25.63) | 14.26 (8.97- 19.81) | 38.72 (23.36- 56) |
| *Different percentiles  for air pollutant^*^* | l = 25^th^ | 16.78 (13.48- 20.17) | 10.75 (6.35- 15.33) | 4.68 (0.35- 9.19) | 26.58 (15.62- 38.59) |
|  | m = 50^th^ | 19.53 (16.64- 22.49) | 14.54 (11.2- 17.98) | 7.44 (4.05- 10.93) | 40.9 (30.48- 52.15) |
|  | h = 75^th^ | 20.92 (18.01- 23.89) | 17.72 (14.5- 21.04) | 10.05 (6.99- 13.19) | 50.03 (38.47- 62.55) |
| *Most populated municipalities (population>43,282 inhabitants)* | l | 10.92 (5.5, 16.62) | 3.23 (-4.41, 11.47) | -1.66 (-8.83, 6.07) | 10.31 (-5.05, 28.15) |
|  | m | 19.38 (15.89, 22.97) | 14.83 (10.84, 18.98) | 7.85 (3.68, 12.19) | 41.44 (29.5, 54.49) |
|  | h | 19.87 (15.89, 23.99) | 21.94 (16.11, 28.06) | 14.97 (9, 21.26) | 53.67 (36.32, 73.23) |
| **O_3_** (μg/m^3^) |  |  |  |  |  |
| *Different definition  of warm period (June-August)* | l | 1.48 (-11.12- 15.87) | -16.77 (-32- 1.87) | -13.61 (-28.84- 4.89) | -20.92 (-54.14- 36.38) |
|  | m | 12.28 (9.29- 15.35) | 16.21 (11.01- 21.65) | 12.76 (6.9- 18.94) | 22.48 (10.65- 35.56) |
|  | h | 11.98 (6.68- 17.55) | 24.8 (14.67- 35.82) | 20.97 (10.68- 32.23) | 34.75 (14.86- 58.09) |
| *Lag 0-3* | l | 19.26 (4.65- 35.92) | 12.21 (-6.59- 34.78) | 8.59 (-14.18- 37.4) | 26.68 (-12.4- 83.21) |
|  | m | 5.07 (1.59- 8.68) | 9.96 (4.74- 15.44) | 10.7 (4.89- 16.84) | 2.36 (-7.05- 12.72) |
|  | h | 11.08 (7.13- 15.17) | 22.63 (15.02- 30.74) | 17.81 (9.5- 26.74) | 39.25 (25.29- 54.76) |
| *Different percentiles  for air pollutant^*^* | l = 25^th^ | 10.32 (4.44- 16.53) | 4.63 (-2.97- 12.81) | 3.24 (-5.48- 12.75) | 0.41 (-18.57- 23.81) |
|  | m = 50^th^ | 11.79 (9.01- 14.64) | 11.56 (6.93- 16.39) | 8.88 (3.72- 14.29) | 12.57 (1.11- 25.33) |
|  | h = 75^th^ | 12.69 (10.4- 15.02) | 17.41 (13.16- 21.83) | 13.22 (8.26- 18.41) | 30.6 (21.09- 40.85) |
| *Most populated municipalities (population>43,282 inhabitants)* | l | 8.76 (-7.05, 27.25) | -5.35 (-22.72, 15.93) | -7.73 (-29.43, 20.66) | 33.97 (-8.07, 95.24) |
|  | m | 12.7 (9.56, 15.93) | 13.04 (7.67, 18.68) | 10.71 (4.97, 16.77) | 18.13 (7.36, 29.99) |
|  | h | 14.3 (9.55, 19.26) | 23.56 (14.61, 33.21) | 16.97 (7.92, 26.78) | 46.8 (27.46, 69.09) |
| **NO_2_** (μg/m^3^) |  |  |  |  |  |
| *Different definition  of warm period (June-August)* | l | 10.75 (6.45- 15.22) | 9.45 (3.6- 15.63) | 2.87 (-3.16- 9.28) | 36.2 (21.18- 53.08) |
|  | m | 14.03 (11.51- 16.61) | 15.28 (12.19- 18.47) | 12.35 (8.62- 16.2) | 29.05 (19.67- 39.16) |
|  | h | 2.18 (-1.43- 5.92) | 3.67 (-1.24- 8.83) | 3.06 (-2.77- 9.24) | 6.85 (-4.12- 19.08) |
| *Lag 0-3* | l | 10.51 (7.38- 13.73) | 5.54 (1.43- 9.82) | 0.13 (-4.07- 4.51) | 19.29 (10.05- 29.31) |
|  | m | 9.63 (7.87- 11.41) | 9.06 (7.18- 10.97) | 6.27 (4- 8.59) | 17.62 (12.5- 22.98) |
|  | h | 7.03 (4.18- 9.96) | 8.23 (4.85- 11.73) | 6.61 (2.97- 10.37) | 7.62 (-2.25- 18.48) |
| *Different percentiles  for air pollutant^*^* | l = 25^th^ | 24.22 (17.19- 31.67) | 14.33 (11.24- 17.51) | 10.91 (7.33- 14.62) | 24.22 (17.19- 31.67) |
|  | m = 50^th^ | 28.16 (19.6- 37.34) | 16.91 (13.9- 19.99) | 13.24 (10.01- 16.55) | 28.16 (19.6- 37.34) |
|  | h = 75^th^ | 21.16 (12.29- 30.73) | 14.84 (11.83- 17.94) | 11.83 (8.84- 14.91) | 21.16 (12.29- 30.73) |
| *Most populated municipalities (population>43,282 inhabitants)* | l | 9.45 (5.96, 13.06) | 7.82 (2.29, 13.65) | 5.48 (-0.95, 12.33) | 13.6 (3.96, 24.13) |
|  | m | 15.59 (13.16, 18.07) | 17.65 (13.77, 21.67) | 13.61 (9.63, 17.73) | 32.7 (21.75, 44.64) |
|  | h | 4.65 (1.14, 8.29) | 6.11 (1.72, 10.69) | 5.86 (-0.06, 12.13) | 1.15 (-10.61, 14.46) |
| Pooled estimates from municipality-specific over-dispersed Poisson regression models, including a tensor smoother for the two-day moving averages of both air temperature and air pollutant, adjusted for seasonal trends and day of the week | | | | | |
